# Monitoring DNA Damage and Repair in Peripheral Blood Mononuclear Cells of Lung Cancer Radiotherapy Patients

**DOI:** 10.3390/cancers12092517

**Published:** 2020-09-04

**Authors:** Pavel N. Lobachevsky, Nicholas W. Bucknell, Joel Mason, Diane Russo, Xiaoyu Yin, Lisa Selbie, David L. Ball, Tomas Kron, Michael Hofman, Shankar Siva, Olga A. Martin

**Affiliations:** 1 Research Division, Peter MacCallum Cancer Centre, Melbourne, VIC 3000, Australia; pavel.lobachevsky@bigpond.com (P.N.L.); joel.mason@unimelb.edu.au (J.M.); drus0009@student.monash.edu (D.R.); yinx1@student.unimelb.edu.au (X.Y.); 2 Advanced Analytical Technologies, Montmorency, VIC 3094, Australia; 3 Department of Radiation Oncology, Peter MacCallum Cancer Centre, Melbourne, VIC 3000, Australia; nick.bucknell@petermac.org (N.W.B.); Lisa.selbie@petermac.org (L.S.); david.ball@petermac.org (D.L.B.); michael.hofman@petermac.org (M.H.); shankar.siva@petermac.org (S.S.); 4 Sir Peter MacCallum Department of Oncology, The University of Melbourne, Melbourne, VIC 3010, Australia; Tomas.Kron@petermac.org; 5 Melbourne Medical School, The University of Melbourne, Melbourne, VIC 3010, Australia; 6 Physics Department, Peter MacCallum Cancer Centre, Melbourne, VIC 3000, Australia

**Keywords:** non-small cell lung cancer, radiation therapy, DNA damage repair

## Abstract

**Simple Summary:**

Every patient responds to radiotherapy in individual manner. Some suffer severe side-effects because of normal tissue toxicity. Their radiosensitivity can be caused by inability of DNA repair system to fix radiation-induced damage. The γ-H2AX assay can detect such deficiency in untransformed primary cells (e.g., peripheral blood mononuclear cells, PBMC), over a period of only hours post ex-vivo irradiation. Earlier we have shown that the level and kinetics of decline (repair) of radiation-induced DNA damage detected by the assay is a measure of the cellular radiosensitivity. In this study, we applied the γ-H2AX assay to judge the radiosensitivity of lung cancer radiotherapy patients as normal or abnormal, based on kinetics of DNA damage repair. Considering the potential of the assay as a clinical biodosimeter, we also monitored DNA damage in serial samples of PBMC during the course of radiotherapy. This study opens an opportunity to monitor individual response to radiotherapy treatment.

**Abstract:**

Thoracic radiotherapy (RT) is required for the curative management of inoperable lung cancer, however, treatment delivery is limited by normal tissue toxicity. Prior studies suggest that using radiation-induced DNA damage response (DDR) in peripheral blood mononuclear cells (PBMC) has potential to predict RT-associated toxicities. We collected PBMC from 38 patients enrolled on a prospective clinical trial who received definitive fractionated RT for non-small cell lung cancer. DDR was measured by automated counting of nuclear γ-H2AX foci in immunofluorescence images. Analysis of samples collected before, during and after RT demonstrated the induction of DNA damage in PBMC collected shortly after RT commenced, however, this damage repaired later. Radiation dose to the tumour and lung contributed to the in vivo induction of γ-H2AX foci. Aliquots of PBMC collected before treatment were also irradiated ex vivo, and γ-H2AX kinetics were analyzed. A trend for increasing of fraction of irreparable DNA damage in patients with higher toxicity grades was revealed. Slow DNA repair in three patients was associated with a combined dysphagia/cough toxicity and was confirmed by elevated in vivo RT-generated irreparable DNA damage. These results warrant inclusion of an assessment of DDR in PBMC in a panel of predictive biomarkers that would identify patients at a higher risk of toxicity.

## 1. Introduction

Radiotherapy (RT) is included in the treatment of half of all patients with cancer. This treatment is commonly used as the primary curative modality in a number of different cancers including carcinomas of the lung, head and neck, cervix and prostate. Across all cancer types, the inclusion of RT is associated with improvements in 5-year local control and overall survival (OS) of an estimated 23% and 6%, respectively [1]. In some tumour types, such as unresectable locally advanced lung cancer, RT is the only available primary curative modality available. The efficacy of RT in cancer treatment is steadily improving, largely driven by rapid developments in technology, which aim to maximize the dose to the tumour while minimizing the dose to normal tissues. The management of cancer is a rapidly evolving landscape with successes including the advent of immunotherapy, targeted therapies, improvements in chemotherapy and surgical oncology. This has resulted in significant improvements in survival (in the United States, there are now >14 million cancer survivors; ~4% of the population) [2,3], such that toxicity remains one of the main concerns when deciding on a treatment regimen.

RT has a prominent role in the treatment of lung cancer. In the curative setting, it is the standard of care in patients with inoperable localized disease and those with mediastinal nodal involvement. In stage III non-small cell lung cancer (NSCLC), RT is the most commonly used primary local treatment. In this cohort the 5-year survival rates are still modest, reported at 32% in a modern RT trial [4]. The recent addition of adjuvant systemic immunotherapy is expected to significantly increase long-term survival, with an ongoing clinical trial reporting an improvement in 2-year OS rate (66% with immunotherapy vs. 56% chemo-RT alone) [5].

Despite advances in technology, RT continues to pose some risk of both acute and late toxicity. Thoracic RT is associated with a risk of developing acute pneumonitis and chronic lung fibrosis [6]. Symptomatic pneumonitis occurs in a third of patients treated with curative intent lung RT, with fatal pneumonitis occurring in 2% of these patients. The prescribed radiation dose and the mean lung dose are the strongest treatment factors associated with pneumonitis [6]. Other factors that increase this risk further include the use of concurrent chemotherapy, the presence of interstitial lung disease and age. There is also a small population of patients who experience significant, even fatal toxicity without any significant risk factors and despite appropriate RT plans. It has been hypothesized that this group of patients may have inherent biological factors underlying their radiation sensitivity (RS), and that if detected before or early in treatment, their plans could be adapted to reduce the risk of toxicity [7].

We conducted a clinical trial that explored gallium-68 ventilation/perfusion (V/Q) positron emission tomography (PET) as an imaging marker of pulmonary toxicity [8]. The overall aim of the trial was to understand the fundamental effects of radiation on pulmonary physiology, to better predict radiation toxicity and to work towards future reductions in radiation toxicity by avoiding irradiation of functionally critical segments of lung. The immune response and accumulated unrepaired systemic DNA damage have been proposed to underlie RT-induced toxicities [9,10,11], and therefore, the associated molecules provide a source of potential predictive biomarkers. Since the relevant tissue for pulmonary toxicity, healthy lung, is inaccessible without unacceptable clinical risk, we restricted our search for predictive biomarkers to minimally invasive blood sampling, concentrating on plasma inflammatory cytokine response to treatment (a manuscript in preparation) and radiation-induced DNA damage response (DDR) in peripheral blood mononuclear cells (PBMC). The latter is a subject of this report.

The discovery in 1998 of histone H2AX phosphorylation (forming γ-H2AX) in response to DNA double-strand breaks (DSB) induction advanced the field of DNA damage and repair [12]. The exquisite sensitivity (a single DSB in a nucleus can be visualized within a nucleus) and speed of DNA damage detection (the signal peaks minutes after the DSB formation) have made the γ-H2AX assay an indispensable tool for measuring DNA damage in situ [13]. The assay has utility across biodosimetry, relative biological effectiveness of novel RT modalities including radionuclide therapy, as well as chemotherapy. One of these exploited the systemic DNA damage propagation in NSCLC RT patients [14]; this study suggested the potential of the accumulated unrepaired DNA damage to predict RT-associated toxicities. Further, in a retrospective study, the assay revealed that compromised DDR is associated with extreme RS [15,16]. Finally, the assay has been used as a biodosimeter in clinical studies [17,18] and has been exploited for clinical trials, e.g., [19,20].

In this study, we followed the γ-H2AX foci formation and disappearance in PBMC collected prior to, during and post-RT treatment in NSCLC patients recruited for the GalliPET VQ-RT prospective clinical trial. We describe a common trend and individual variations of DDR to the treatment. We searched for associations of the observed DDR with various types of RT-related toxicity and the tumour response.

## 2. Results

**Figure 1:**
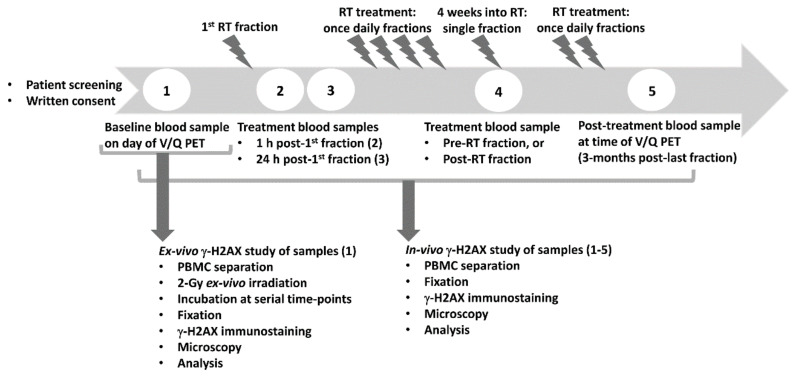
Experimental scheme of the study. Patients with non-small cell lung cancer (NSCLC) were recruited for the GalliPET VQ-RT clinical trial. The γ-H2AX biomarker study was a biological part of the clinical trial. Venous blood was collected at the indicated time points before, during and after radiotherapy (RT) treatment and processed as described in Section 4. Peripheral blood mononuclear cells (PBMC) collected at all time points were not exposed to ex vivo irradiation (in vivo γ-H2AX study), except an aliquot of a baseline PBMC sample that was subsequently ex vivo irradiated (ex vivo γ-H2AX study). Ventilation/perfusion (V/Q) positron emission tomography (PET) prior to treatment and post-treatment enabled assessment of treatment toxicity and tumour progression.

Experimental scheme of the study is presented in Figure 1. We collected blood samples before commencement of RT (“baseline”), at 1 h post first RT fraction, 24 h post first RT fraction before the 2nd RT session, 4 weeks into RT and 3 months after the end of RT. The study consists of two parts: (1) in vivo study, where PBMC were not exposed to ex vivo irradiation, thus detected γ-H2AX foci per cell (fpc) that were induced either by endogenous factors or by RT treatment for NSCLC and (2) ex vivo study, where PBMC collected prior to commencement of RT were exposed ex vivo to 2-Gy irradiation that induced the γ-H2AX response.

### 2.1. In Vivo Study: γ-H2AX Foci in PBMC that Were Not Exposed to Ex Vivo Irradiation

Isolated PBMC were fixed immediately after blood collection (and subsequently immunostained for γ-H2AX) and, when it was logistically possible, also at 24 h post blood collection. Table 1 summarizes the statistics of fpc numbers for each group in PBMC that were fixed early: the arithmetic mean value, standard deviation, number of samples, and *p*-values for the comparison of each group with the baseline. Table S1 shows the same values for PBMC that were fixed 24 h after blood collection. The 4-week time point was a mixed group. Due to treatment logistics, i.e., timing of patients’ RT and chemotherapy schedules, some blood samples were collected after delivery of the RT session (at 1–6 h), while some were collected before the session. The results for each of these two subgroups are shown separately in Table 1.

**Figure 2:**
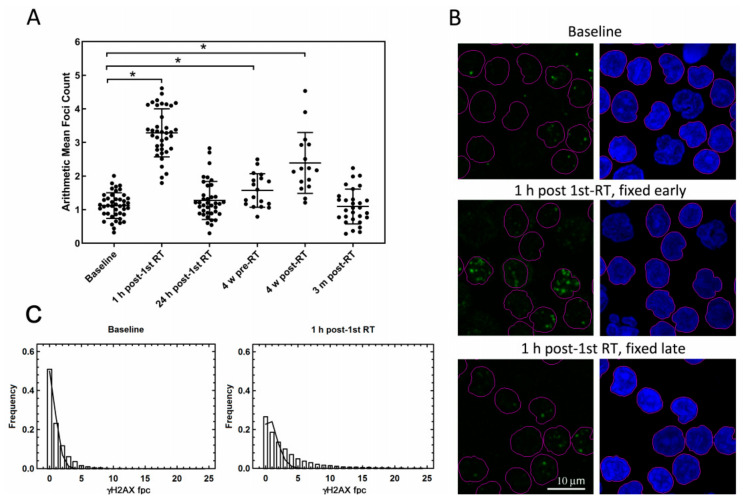
γ-H2AX foci in PBMC not exposed to ex vivo irradiation. (**A**) γ-H2AX foci counts in PBMC for all time points and patients (PBMC were fixed immediately after blood collection). Scatter plots of γ-H2AX foci numbers from groups of samples obtained at different time points of radiotherapy (RT) treatment. For data obtained from samples collected at 4 weeks into RT, two subgroups are shown, samples obtained pre-RT session and post-RT session, as explained in the text. Each point represents the arithmetic mean of foci numbers counted in images from an individual patient. Bars show the mean values and standard deviations for each group. * Statistically significant. (**B**) Representative confocal microscopy images of PBMC immunostained with anti-γ-H2AX antibody from patient GP39 at baseline (top row), 1 h after the first RT session when PBMC were either fixed immediately after blood collection (fixed early; middle row) and when PBMC were fixed 24 h after blood collection (fixed late; bottom row). The left column shows green channel, γ-H2AX, and right column shows blue (DAPI) channel (nuclei). Objects (nuclei) selected for the analysis are outlined in pink. Scale bar, 10 μm. (**C**) Averaged γ-H2AX foci frequency distributions in PBMC collected at baseline (left panel) and 1 h post first RT (right panel). Solid lines are the best fit Poisson distributions. Arithmetic mean values are 1.08 and 3.18, best-fit Poisson average values 0.53 and 1.06 and R-squared values for the nonlinear regression are 0.975 and 0.853, for the baseline and 1-h groups, respectively.

Figure 2A illustrates dynamics of γ-H2AX fpc in PBMC that were fixed immediately after blood collection, for all time points and patients, and representative microscopy images are shown in Figure 2B. There is a significant increase of mean foci numbers at two time points of blood collection, approximately three fold at 1 h post first RT session (3.24 fpc) compared to the baseline value (1.11 fpc), *p* < 0.0001, for both paired and unpaired *t*-test, and at 4 weeks in the RT, with more than two fold increase in samples collected post-RT session (2.39 fpc, *p* < 0.0001 for both paired and unpaired *t*-test), and some increase in samples collected pre-RT session (1.57 fpc, *p* = 0.0089, paired *t*-test). The substantial increase of foci post-RT sessions is assumed to be related to the presence in the blood samples of cells that had been in the irradiated volume during delivery of RT treatment [14], as described in details in Text S1. This interpretation is consistent with the subsequent decrease of fpc in the same samples that were fixed late, at 24 h post blood collection (Table S1), thus allowing time for foci elimination as a result of γ-H2AX dephosphorylation, signifying ex vivo repair of radiation-induced DSB. A similar decrease of fpc was observed in the samples collected at 24 h post first RT fraction (Table 1), ascribed to in vivo repair.

The analysis of foci frequency distributions (Figure 2C) supports this interpretation. The background foci form randomly, and their distribution follows Poisson statistics, as indicated by high R-squared value of nonlinear regression analysis (0.975). If a subpopulation of cells is irradiated, a two-component foci frequency distribution is expected. Indeed, at 1 h post first RT session, apart from the higher mean foci number, the agreement of the experimental histogram with Poisson distribution is weaker (*R* = 0.853), due to the presence of cells with a substantially higher foci numbers than expected from the random distribution. A further analysis of foci frequency distributions is presented in the first paragraph of Section 2.3.

### 2.2. Ex Vivo Study: Kinetics of γ-H2AX Foci in Baseline PBMC that Were Exposed to Ex Vivo Irradiation

**Figure 3:**
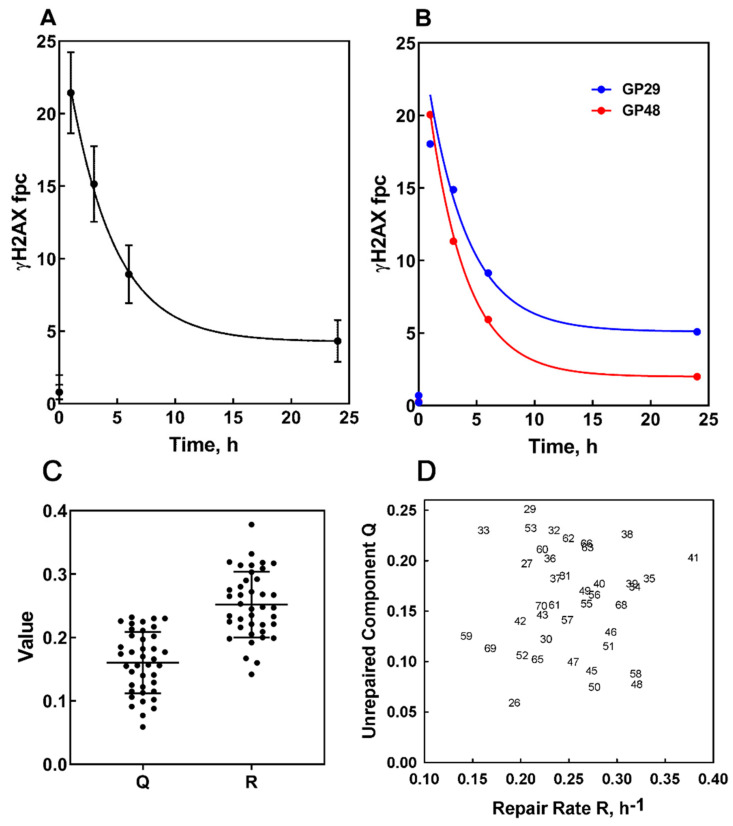
γ-H2AX foci kinetics following ex vivo irradiation of PBMC collected at baseline. PBMC were collected prior to RT, exposed to 2 Gy-irradiation and fixed at serial time points. (**A**) Average γ-H2AX foci kinetics for all patients. Data points and error bars represent the mean foci number across all patients and the standard deviation. (**B**) Representative examples of a large variation of foci kinetics between patients; with small *Q* (the fraction of irreparable DNA damage) and large *R* (the fraction of fpc per hour) (GP48) and large *Q* and small *R* (GP29). The symbols represent experimental points, and the lines are obtained as a result of nonlinear regression. Values of the best fit parameters *Q* and *R* are 0.225 ± 0.0624 and 0.199 ± 0.060, respectively, for GP29 and 0.077 ± 0.011 and 0.319 ± 0.018, respectively, for GP48. (**C**) Scatter plots of *Q* and *R* values obtained from nonlinear regression of foci kinetics. Each point on scatter diagrams represents an individual patient. Mean values ± standard deviations are 0.163 ± 0.046 and 0.254 ± 0.052 for *Q* and *R* values, respectively. (**D**) *Q-R* map (“RS map”) showing values of the parameters in *R* and *Q* coordinates. Each point represents an individual patient, and numerical values correspond to the patient numbers. The top left-hand region of the map corresponds to compromised DNA repair.

Aliquots of PMBC from blood samples collected at baseline (before commencement of RT) were irradiated ex vivo to a dose of 2 Gy ^137^Cs γ-rays, or 320 KeV X-rays, and fixed at serial time points (1, 3, 6 and 24 h). In Figure 3A,B regression analysis of post irradiation γ-H2AX foci kinetics averaged for the entire cohort is shown (Figure 3A), as well as representative examples of a large variation between patients (Figure 3B).

We previously reported that two parameters derived from the nonlinear regression analysis (curve fitting) of foci kinetics (Equation (4) from Section 4), as well as their combination, were associated with extreme normal tissue toxicity in RT patients [15]. The two parameters reflect the fraction of DNA damage, which is apparently irreparable, determined by extrapolation to infinite time, of the curve fitted through the data points for each plot following the decay of damage with time (*Q*) and the rate of repair as a fraction of fpc per hour (*R*). The *Q* and *R* are individual for each dataset (i.e., each patient); the values reported here correspond to the statistically best fit curves through the experimental data. The best predictor of extreme RS was the combination of these two values, the 2-D mapping onto *R* and *Q* coordinates (“RS map”) [15]. The results of the foci kinetics analysis are summarized in Table S2, and displayed in Figure 3C,D. Figure 3C shows scatter plots of *Q* and *R* values, and Figure 3D shows the RS map for all patients. The frequency distributions for both values (shown in Figure S1) pass the normality tests (*p* > 0.1, Kolmogorov-Smirnov test), indicating that each data set is derived from a single population without obvious outliers.

**Figure 4:**
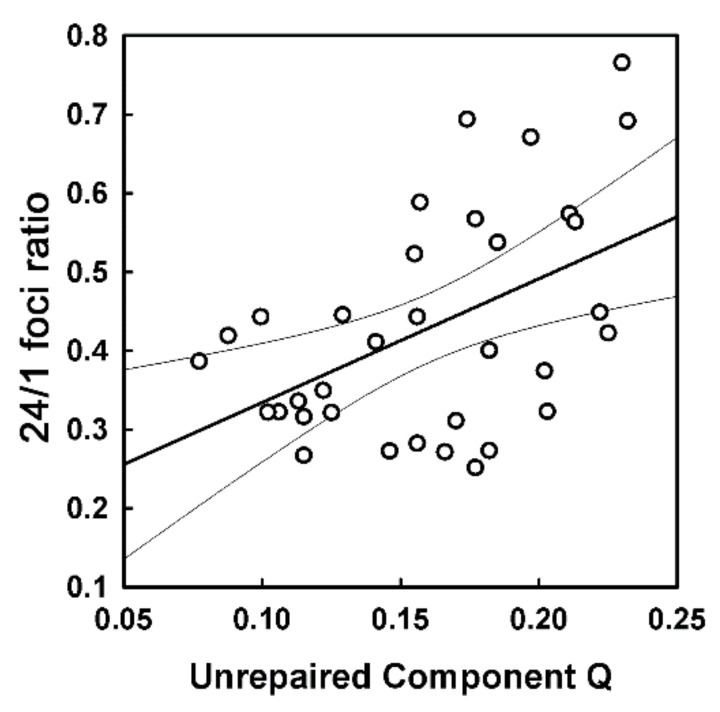
Correlation between *Q*-values and the ratio of background foci in PBMC collected at 24 and 1 h post first radiotherapy (RT) session. The 24/1 h ratios for all patients are presented in Table S2. This ratio reflects the efficiency of in vivo repair of DNA damage induced in PBMC by RT treatment.

To understand if DSB repair in ex vivo irradiated PBMC correlates with DSB repair in PBMC exposed to RT in vivo, we studied a correlation between *Q*-values and the ratio of foci in PBMC collected at 24 and 1 h post first RT (shown in Table S2). The lower ratio indicates better repair. We found a statistically significant correlation between these values (*r* = 0.487, *p* = 0.0035), as illustrated in Figure 4. We also found a statistically significant correlation between fpc numbers in PBMC collected at 24 h post first RT session and *Q*-values (*r* = 0.529, *p* = 0.001).

### 2.3. Correlation Studies with Treatment Conditions; PBMC that Were not Exposed to Ex Vivo Irradiation

We further deconvoluted γ-H2AX foci that originated from RT and background foci, by applying a nonlinear regression analysis to foci frequency distributions (Equation (1) from Section 4). Nonirradiated PBMC have random background foci that follow Poisson statistics (Equation (2) from Section 4). Radiation-induced foci are generally random events as well, but foci induced in a fraction of PBMC that were exposed while traveling through the irradiated thorax and receiving different doses, do not follow Poisson statistics. For these cells, we used gamma-distribution (Equation (3) from Section 4). The best fit values were obtained for the fraction of irradiated cells and the average number of radiation-induced foci in these cells. The results of this analysis for each patient are presented in Table S3.

**Figure 5:**
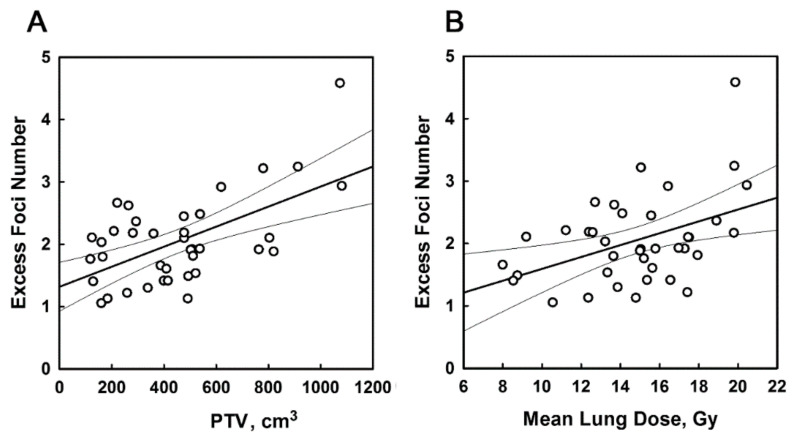
Correlation of the excess foci number with planned treatment volume (PTV) and mean lung dose (MLD). (**A**) PTV and (**B**) MLD correlation analysis for a group of patients with blood samples collected at 1 h after first RT session. Each point represents the data for an individual patient. The excess foci number is calculated as the product of the average radiation-induced fpc and the fraction of irradiated cells from Table S3. The lines show the linear regression and 95% confidence intervals. Correlation coefficient *r* = 0.586, *p* = 0.00011 for PTV and *r* = 0.437, *p* = 0.0006 for MLD.

These values were used in the correlation analysis with planned treatment volume (PTV) and mean lung dose (MLD). A weak association of the average fpc numbers with PTV (*r* = 0.235, *p* = 0.155) was observed. A statistically significant correlation was found between the fraction of irradiated PBMC and PTV (*r* = 0.417, *p* = 0.009). The product of the average radiation-induced fpc number and the fraction of irradiated PBMC demonstrated the best correlation with PTV (*r* = 0.586, *p* = 0.0001). This product equals to the excess foci number that is defined as a difference between mean fpc in PBMC collected at 1 h post first RT fraction and at baseline. The results are summarized in Table 2 and illustrated in Figure 5A.

We found a weaker association of the excess foci number with MLD (Table 2 and Figure 5B; *r* = 0.437, *p* = 0.006). MLD correlated significantly with PTV (*r* = 0.597, *p* < 0.0001), thus these values are interdependent. We also generated a combined PTV and MLD variable as a linear combination of these values that provides the best fit for the excess foci number. The excess foci number correlated with this combined variable better than with PTV or MLD (*r* = 0.601, *p* < 0.0001) indicating that the dose to both tumour and lung contributes to the induction of foci (Table 2).

We also calculated the number of RT-induced foci by taking into account PTV and MLD, as described in Text S1. Although, due to limited accurate information on blood flow in the thorax and treatment duration, it is difficult to obtain a reliable prediction of the fraction of irradiated cells and the average foci number, it is possible to establish the relationship between these values, using average values for PTV (500 cm^3^) and MLD (0.486 Gy/fraction). The results are shown in Figure S2. The average foci numbers obtained from experimental data (Table S3) correlated with calculated foci numbers (*r* = 0.604, *p* < 0.01). The values were calculated as described in Text S1 using treatment parameters for each patient, however, the calculated values underestimate the experimental values. This analysis also indicates that irradiation of both tumour and lung contributes to the induction of foci in PBMC to a similar extent, with average per irradiated cell doses of 33 and 49 mGy for tumour and lung, respectively.

### 2.4. Correlation Studies with Treatment Outcomes; Baseline PBMC that Were Exposed to Ex Vivo Irradiation

We explored whether an association exists between the unrepairable component *Q* and repair rate *R* values across 7 types of RT-induced acute toxicity using CTCAE criteria. The severity of each type of toxicity was evaluated as a four-level grade score (0–3; there were no treatment-related CTCAE grade 4 or 5 toxicities in the cohort). For each patient, we first calculated the combined toxicity grade (the sum of grades for each type of toxicity, which varied from 3 to 11) and found a weak association of this parameter with *Q*-values (*r* = 0.152, *p* = 0.36, Figure S3). Then, we applied the *t*-test to compare *Q*-values in 3 subgroups of patients with different toxicity grades for each of the 7 types of toxicity. These subgroups corresponded to toxicity grades 0, 1 and combined 2 and 3, since only 5 cases of grade 3 toxicity were observed. The results including average values for each subgroup and *p*-values for comparison of grades 1 and 2 with grade 0 are summarised in Table 3. Scatter plots illustrating this analysis are presented in Figure S4.

**Figure 6:**
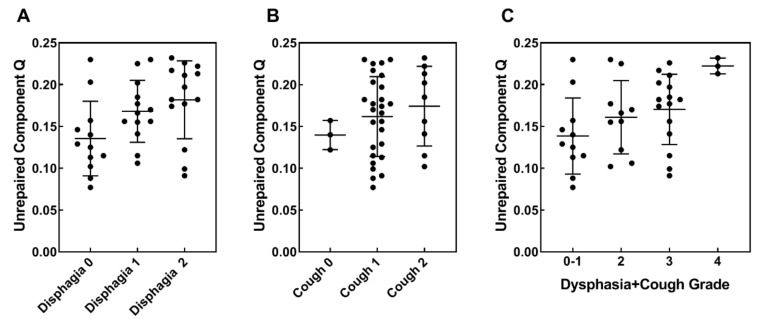
Comparison of *Q*-values in subgroups of patients with acute normal tissue toxicity—Dysphagia and cough. Diagrams show scatter plots of *Q*-values in subgroups of patients with: (**A**) grades 0, 1 and 2 of dysphagia; (**B**) grades 0, 1 and 2 of cough; (**C**) combined grades of dysphagia and cough. Mean *Q*-values and their standard deviations for each subgroup are presented in Table 3.

For two types of toxicity, dysphagia and cough, a trend for increasing *Q*-values in subgroups with higher toxicity grades was observed. For the increase of *Q*-values for grade 1 and 2 cough, *p*-values were 0.155 and 0.098, respectively; for grade 1 dysphagia, *p* = 0.061 and statistically significant for grade 2 dysphagia (*p* = 0.016). Scatter plots illustrating this trend are presented in Figure 6A,B.

To look further into the relationship between *Q*-values and the severity of dysphagia and cough, we calculated the combined criterion as a sum of dysphagia and cough toxicity grades and analyzed four subgroups (grades 0–1, 2, 3 and 4). A similar trend for increasing *Q*-values was observed for this combined criterion with a statistically significant increase in grade 4 compared to grade 0–1 (*p* < 0.001) (Figure 6C). A positive correlation was found between *Q* and the combined dysphagia/cough toxicity grade (*r* = 0.412, *p* = 0.010, Figure S3). These results indicate that dysphagia and cough are not independent toxicities.

The analysis of the combined dysphagia/cough toxicity identified three patients with combined score 4 (GP53, GP62 and GP63) that have *Q*-values at the highest end of the range (0.21–0.23). Interestingly, for patients GP53 and GP62, the toxicity grade 2 was observed for 5 out of 7 toxicity criteria, including radiation-induced pneumonitis, and only for these patients, the overall combined toxicity grade reached 11. These patients map to the higher RS zone on the 2-D RS map (Figure 3D) and correspond to higher ratios of mean foci number in samples collected at 24 h vs. 1 h post first RT, reflecting compromised efficiency of the repair of RT-induced foci. This ratio is higher for GP53, GP62 and GP63 (0.69, 0.45 and 0.56, respectively) than the average ratio of 0.41 (Table S2). On a retrospective chart review for the presence of late toxicity (median follow up time 2 years), all three of these patients had unusual pulmonary toxicity, persistent grade 2 symptoms of cough and/or shortness of breath, and ultimately required ongoing medical interventions. All patients also demonstrated a complete metabolic response at 3 months post-treatment. At the time of last follow up, all patients were alive and only one patient developed a recurrence, which was able to be salvaged with curative intent.

No correlation was found between normal toxicity grades and values of repair rate R (Figure S5), as well as between metabolic response and both *Q* and *R* values, as evidenced from the data presented in Table 4.

## 3. Discussion

Contemporary practice in thoracic radiation oncology involves initially prescribing the established curative regime of 60–66 Gy in 30–33 fractions for all patients and planning the RT to determine if the volume can be safely treated with the prescribed dose. A complex interaction between radiation-induced damage to parenchymal cells, supporting vasculature and associated fibrotic reactions results in acute and late radiation toxicities. In the lung, these changes can manifest in reduced pulmonary function and in a chronic inflammatory cascade, which results in pneumonitis [21].

To date, the strongest predictors of radiation-induced lung injury are based on the volume of normal lung tissue irradiated. These parameters (V20 and MLD) have been validated on meta-analysis of multiple thoracic RT trials, and are often used as binary limits to select patients for the ability to tolerate curative intent treatment with an acceptable toxicity profile [6]. There are, however, a population of patients that experience significant toxicity without any clinical risk factors and with acceptable RT dosimetric parameters. It has been hypothesised that such patients may have inherent biological factors underlying their RS and that if detected with an assay before or early in course of RT, their treatment plans could be adapted to reduce risk of toxicity. The ideal biomarker would identify patients at higher risk of toxicity while also providing information on the likelihood of tumour response to treatment. A biomarker, if validated, would enhance the informed consent process where patients could be more accurately advised of the probability of developing treatment-related toxicity and of tumour control for a given prescribed dose. This would foster engagement in the process of planning and empower patients to have a greater say in their treatment.

A number of strategies have been proposed for such advances in personalized care in thoracic radiation oncology. These include the use of functional lung imaging to identify and avoid regions of working lung during the planning process, the use of early and mid-treatment cytokine levels to identify patients at increased risk of lung toxicity and the use of novel biomarkers to predict toxicity risk [11,22,23]. Previously, we have demonstrated that RT-related early changes in a panel of plasma cytokines were associated with higher grades of radiation-induced lung toxicity [23]. Changes in concentrations of IP-10, MCP-1, Eotaxin, IL-6 and TIMP-1 have also been associated with accumulation of DNA damage in normal tissues outside of the irradiated volume during RT treatment for NSCLC [14].

In a retrospective case-study of a patient with severe clinical RS, our group demonstrated inefficient repair of DNA damage, as assessed by the kinetics of the γ-H2AX response, in PBMC and hair follicles, following ex vivo irradiation [24]. This led to a more comprehensive retrospective study from which statistical criteria were proposed to define DNA repair deficiency [15,16]. However, we have subsequently found that RT itself modulates the repair capacity [25]. Thus, we can anticipate that the counterpart of the RS map published in our retrospective study [15], which would emerge from a prospective study, would have modified scales. The qualitative features of the map would be retained, e.g., the points for radiosensitive individuals would map the region in the top left-hand corner. In this context, the RS map has been helpful in the current study. More generally, a large prospective study will be required to refine our statistical criteria of severe clinical RS for the γ-H2AX-based predictive assay.

The published prospective studies that utilized γ-H2AX as a predictive biomarker for both acute and late normal tissue toxicity revealed mixed results. In clinical trials aiming to prospectively identify head and neck cancer RT patients with a higher degree of radiation-induced mucositis, Fleckenstein et al. [26] found a trend for delayed DSB repair. Similarly, Goutham et al. [27] and Li et al. [28] successfully separated overresponding and non-overresponding patients based on post irradiation γ-H2AX kinetics, and in one of these studies [27], also for residual foci. On the other hand, in a number of clinical studies, the assay failed to predict RS in prospective and retrospective patient cohorts, e.g., [29,30,31]. It is worthwhile to note that in some of the negative reports, the failure could be ascribed to suboptimal study design and analysis. None of the clinical RS studies published to date involve lung cancer RT patients. The GalliPET VQ-RT clinical trial that has been conducted at our institute provided an opportunity to study real-time dynamics of DNA damage accumulation and resolution in PBMC of 38 NSCLC patients that were collected before, during and after RT treatment and to associate the individual DDR with the patients’ clinical responses.

We found a substantial increase in the mean number of γ-H2AX foci in PBMC collected from patients at 1 h post first RT session compared to pre-treatment values. We concluded that these foci were induced by irradiation of cells while they were traveling within the RT treatment volume. The analysis of foci frequency distributions based on the proposed model allowed calculation of the fraction of cells subjected to irradiation and the average and excess foci number in these cells. The excess foci number correlated with PTV, MLD and a combined PTV/MLD parameter. This study indicated that irradiation of blood in both tumour and normal lung tissue is a source of DNA damage in PBMC. The numbers of induced foci obtained from experimental distributions exceeds those predicted from calculation based on RT treatment parameters, indicating that potentially substantial DNA damage may emerge from irradiation of other than lung normal tissues that are located within the irradiation beam.

An increased mean foci number was also observed in PBMC collected 4 weeks into RT. Given treatment logistics necessitated that some 4-week samples were collected before RT treatment and some were collected after RT treatment; we analyzed these two subgroups separately. A substantial and statistically significant increase of the mean foci number was found in samples collected after RT treatment, supporting the interpretation that irradiation of cells within the treatment volume accounts for these foci. A smaller, yet still statistically significant, increase of the mean foci number was also found in the subgroup of samples collected before the RT fraction in the 4-week group. This could be explained by induction of DNA damage by systemic (abscopal) effects in susceptible progenitor cells that is revealed later as increased unrepaired damage in PMBC [17,32].

In the study of ex vivo 2 Gy-irradiated PBMC, we considered the unrepairable component *Q*, the value derived from the nonlinear regression analysis of post irradiation foci kinetics, as an indicator of the efficiency of DNA repair and a predictor of radiotoxicity [15]. We found no obvious outliers in a set of *Q*-values, therefore, no patients predicted to have severe RS were revealed. In parallel with this finding, there were no unusually severe clinical toxicities in the patient cohort. However, evidence from preclinical and clinical observations suggests that normal tissue toxicity is multifactorial, dynamic and progressive process [33,34,35]. Accordingly, in subgroups of patients with various grades of radiotoxicity, we found a correlation between *Q*-values and the toxicity grade for cough and dysphagia. *Q*-values and the position in the 2-D RS map were in the high RS zone for three patients with the highest combined cough and dysphagia grade. These patients also demonstrated other types of toxicity and had persistent health problems that required clinical intervention. They demonstrated a complete metabolic response, suggesting that their tumours were also sensitive to radiation.

We also considered the ratio of the mean foci number in samples collected 24 and 1 h after the first RT session, which reflects the in vivo repair of DNA damage induced by RT. We found a good concordance of this ratio with *Q*-values (Figure 4). Indeed, the three patients with high grade of cough/dysphagia had higher than average ratio values, further supporting this concordance. Despite this trend, however, the ratio is not as accurate as *Q*-value, hence no statistically significant correlation between patient subgroups with different levels of toxicities was found, except for the trend of the increased ratio with the increased combined cough and dysphagia toxicity (similar to the foci numbers in PBMC collected at 24 h post first RT session). These values are simpler parameters than the in vitro *Q*-value and can be used as a triage, to reveal patients that are potential candidates for the more precise ex vivo irradiation test. Their accuracy could be improved by increasing the number of analyzed cells and by standardizing immunostaining/microscopy protocols to achieve high image reproducibility.

## 4. Materials and Methods

### 4.1. Patient Recruitment

Patients with NSCLC (*n* = 45) were recruited for the GalliPET VQ-RT clinical trial (ethics approval from the Peter MacCallum Cancer Centre; Universal Trial Number U1111-1138-4421, date of approval 7 July 2014). The patients were aged between 49 and 82 years (the median age was 68); 30 males and 15 females. A total dose of 60 Gy was delivered to all patients in 30 fractions over 6 weeks using a 3D conformal technique. Six patients received RT only; 39 received RT with concurrent chemotherapy. All patients gave written informed consent for participation in this trial. Patient information is presented in Table 4. Toxicities were recorded using Common Terminology Criteria for Adverse Events (CTCAE) v4.0. We did not assume that the gallium-68 perfusion protocol with the whole-body radiation dose of the PET scan ~50 mSv could interfere with the results of the study. The impact was controlled as all patients were exposed to the GalliPET protocol.

### 4.2. Blood Collection and Processing

Blood samples from 43 patients were analysed. Of the 45 recruited trial patients, 7 did not complete the full course of treatment (due to disease progression), changed to palliative treatment due to the extent of disease or lacked a full γ-H2AX dataset due to sample loss or processing failures. A complete dataset with a full complement of translational biomarker time points was, therefore, available for 38 patients.

The logistic of the study is presented in Figure 1. Venous blood (15 mL) was collected in ethylenediaminetetraacetic acid (EDTA) tubes at each time point: pre-treatment (baseline), 1 h after the first RT fraction, approximately 24 h after the first RT fraction (just before the second fraction), 4 weeks into the treatment course (before or after RT fraction) and 3 months after the final RT treatment (at the follow-up appointment). The protocol for blood processing has been described previously, i.e., [15]. As soon as possible after blood collection, PBMC were separated by centrifugation over Ficoll-Paque PLUS (GE Healthcare Life Sciences, Sheffeld, UK) at 600*× g* for 45 min (without brakes) and washed in the in-house phosphate buffered saline (PBS). Aliquots of isolated PBMC were fixed immediately post blood collection and also aliquots of the majority of PBMC samples were fixed at 24 h post blood collection.

Aliquots of PBMC from all baseline samples were incubated for 30 min at 37 °C in RPMI-1640 medium supplemented with 10% foetal bovine serum (Gibco Life Technologies, Mulgrave, VIC, Australia) and 0.1% gentamicin (Pfizer, Sydney, NSW, Australia). PBMC were then transferred into 15 mL tubes filled with fresh warm complete medium and irradiated to a dose of 2 Gy. Due to relocation of the research laboratory, initially a ^137^caesium source (GammaCell 40 Irradiator, Nordion International, Kanata, ON, Canada) at a dose-rate of 0.53 Gy/min, and later X-rays (X-RAD320 Irradiator, Precision X-ray, North Branford, CT, USA) at a dose-rate of 1.15 Gy/min, were used for irradiations. The two radiation sources were compared with respect to the γ-H2AX response in ex vivo 2-Gy irradiated PBMC. Fricke dosimetry was used in the comparison studies. The cells were then transferred to 25 cm 2-tissue culture flasks, incubated at 37 °C in 5% CO_2_ in air, sampled at 1, 3, 6, and 24 h post irradiation, fixed in 4% paraformaldehyde, washed in PBS and stored in 70% ethanol at −20 °C.

In addition, PBMC from all collected samples including baseline that were not exposed to ex vivo irradiation were fixed immediately after blood collection, and, when it was logistically possible, other aliquots were incubated overnight in the complete medium and subsequently fixed at 24 h postblood collection. All PBMC samples were stored in 70% ethanol at −20 °C until immunostaining.

### 4.3. Immunostaining, Microscopy and Image Analysis

PBMC were cytospun onto microscope slides (Menzel-Glaser Lomb, Braunscheig, Germany) using a Cytospin 4 cytocentrifuge (Thermo-Scientific, Waltham, MA, USA) for 4 min at 800*× g*. The cell spots were dried for 10 min, outlined with a PAP pen (ProSciTech, Kirwan, QLD, Australia) and blocked with 8% BSA (Sigma-Aldrich, St Louis, MO, USA) for 30 min prior to immunostaining. The slides were incubated with a mouse monoclonal anti-γ-H2AX antibody (Abcam, Cambridge, UK, 1:500) in a humidified chamber at room temperature for 2 h, then after washing, cells with PBS were incubated with Alexa Fluor 488-conjugated goat anti-mouse IgG secondary antibody (Invitrogen, Mulgrave, VIC, Australia, 1:500) for 1 h. Primary and secondary antibodies were diluted into 1% BSA in PBS-TT (PBS containing 0.5% Tween-20 and 0.1% Triton X-100, Sigma-Aldrich, St Louis, MO, USA). Slides were washed three times with PBS and mounted in 4′,6′-diamidino-2-phenylindole (DAPI)-containing Vectashield mounting medium (Vector Laboratories, Burlingame, CA, USA).

Microscopy was performed with an Olympus FV1000 laser confocal scanning microscope (Olympus, Tokyo, Japan). Serial optical sections were used to create a single maximum projection image for each field; these images contained all detected γ-H2AX foci throughout the nuclear volume. A minimum of 3 fields were analysed for each time/dose point; usually sufficient to count foci in at least 100 cells. The nuclei of mononuclear cells were identified, and γ-H2AX foci within these nuclei were counted automatically using the in-house developed software JQuantPro v2.0 [36,37].

### 4.4. Data Analysis

#### 4.4.1. Distributions of Foci Numbers

We analysed foci frequency distribution for the sole purpose of evaluating the fraction of irradiated cells and the average number of radiation-induced foci in these cells. Distributions of foci frequencies in untreated PBMC from samples collected at 1 h post first fraction of RT were approximated by least squares regression using the following expression for the probability of a cell to have *k* foci:

(1) $$P_{k}\left(\lambda ,\alpha ,\beta ,f\right)=\left(1-f\right)P_{k}^{P}\left(\lambda \right)+f\overset{k}{\underset{i=0}{\sum }}P_{i}^{P}\left(a\right)G_{k-i}\left(\alpha ,\beta \right)$$

which assumes that γ-H2AX foci originate from two sources, randomly distributed background foci and foci that are induced by irradiation in PBMC, which are located within the irradiation volume in the course of RT. The parameter *f* denotes the fraction of PBMC that are subject to irradiation. The count of background foci is assumed to follow a Poisson distribution with average value of *λ*:

(2) $$P_{k}^{P}\left(\lambda \right)=e^{-\lambda }\frac{\lambda^{k}}{k!}$$

and the number of radiation-induced foci are assumed to follow a gamma distribution with parameters *α* and *β*:

(3) $$G_{k}\left(\alpha ,\beta \right)=\frac{\beta^{\propto }}{\Gamma \left(\alpha \right)}k^{\alpha -1}e^{-\beta k}$$

The purpose of least squares regression is to evaluate the fraction of irradiated PBMC (*f*) and the average number of induced foci in these cells (calculated as the ratio *α*/*β*). Gamma distribution is chosen as an empirical model suitable for the description of the distribution of foci in cells following irradiation with a range of doses without any implied biological background.

#### 4.4.2. Analysis of Foci Kinetics

For the analysis of γ-H2AX kinetics in PBMC irradiated ex vivo with 2 Gy X-rays, we first calculated the modal average foci number values for each time point t (denoted below as *N*(*t*)) by applying least squares regression according to Equation (2) to the experimental foci frequency distributions. As we demonstrated previously, the Poisson modal parameter estimate provides a better description of the count of radiation-induced foci than an arithmetic mean. The kinetics of foci elimination was analysed using a least square with a model that assumes the exponential repair of DSB with a rate *R* and the presence of irreparable component *Q* as unknown parameters, according to the following equation:

(4) $$N\left(t\right)=N_{m}\left(\left(1-Q\right)e^{-Rt}+Q\right)$$

### 4.5. Statistical Analysis

Paired or unpaired *t*-tests were used for the comparison of patient foci numbers between groups. For correlation studies, we used Pearson product moment correlation (correlation coefficient r and corresponding *p*-value).

## 5. Conclusions

This study provided novel information on the clinical utility of the γ-H2AX assay to monitor DDR in a clinical setting. Our earlier retrospective studies [15,16] provided a starting point towards the objective of developing an assay to prospectively identify radiosensitive patients using the γ-H2AX-based assay to assess the efficiency of their DNA repair. The current study of 38 patients provided valuable information. The three NSCLC patients who scored highly on the assay could not be described as extremely radiosensitive, at least in the time-frame of 36 months between treatment and the present time. However, as survival in locally advanced NSCLC is poor, long-term survivorship and development of very late toxicities are rare. Based on our results, in a large prospective trial, we can expect that the number of patients with clinically proven normal tissue toxicities would be less than the number found to have compromised DNA repair, indicating the requirement for a more sophisticated approach to predict RS. This could be achieved by combining the γ-H2AX assay with other functional assays that showed a predictive potential, such as initial DNA damage assessment, e.g., ATM nucleo-shuttling [38], radiation-induced lymphocyte apoptosis (RILA) assay [39], or chromosome aberrations [40]. Nevertheless, the detection of compromised DNA repair in the three patients showing modest clinical RS, and requiring special follow-up, raises the possibility of prospective identification of modestly radiosensitive patients that could be of clinical benefit.

## Figures and Tables

**Table 1:** Statistics of γ-H2AX foci per cell (fpc) values in peripheral blood mononuclear cells (PBMC) collected at different time points and fixed immediately after blood collection.

Time Points of Blood Collection	Baseline	1 H Post First RT	24 H Post First RT	4 Weeks ^†^ (Sample Pre-RT)	4 Weeks ^†^ (Sample Post-RT)	3 Months Post-Final RT
Mean Value, fpc	1.11	3.24	1.26	1.57	2.39	1.09
Standard Deviation	0.37	0.68	0.57	0.50	0.91	0.52
Number of samples	43	38	38	18	16	29
*p*-value ^1^ relative to baseline		<0.0001 *	0.130	0.0089 *	<0.0001 *	0.688

^1^*p*-values are given for paired *t*-test. * Statistically significant. ^†^ Due to treatment logistics some samples taken at 4 weeks were collected before the RT session (pre-RT) and some were collected after (post-RT).

**Table 2:** Pearson correlation of foci induction at 1 h post first radiotherapy (RT) with planned treatment volume (PTV) and mean lung dose (MLD).

Parameter	PTV		MLD		Combined PTV and MLD	
	Correlation Coefficient	*p*-Value	Correlation Coefficient	*p*-Value	Correlation Coefficient	*p*-Value
Average per cell number of induced foci	0.235	0.155	0.234	0.158		
Fraction of affected cells	0.417	0.010	0.274	0.0955		
Average per cell number of excess foci	0.586	0.00011	0.437	0.00060	0.601	<0.0001
MLD	0.597	<0.0001				

**Table 3:** Summary of the statistics and comparison of *Q*- and *R*-values for subgroups of patients with various metabolic response and normal tissue toxicity grades for seven types of toxicity.

Tumour Response/Toxicity	Grade	*N*	*Q*-Values			*R*-Values		
			Average	SD	*p*-Value	Average	SD	*p*-Value
Tumour response	CMR ^1^	12	0.155	0.048		0.246	0.048	
	PMR ^2^	10	0.159	0.049	0.827	0.251	0.061	0.821
	PMD ^3^	7	0.167	0.048	0.600	0.277	0.039	0.146
Dyspnoea	0	7	0.161	0.044		0.226	0.050	
	1	18	0.175	0.043	0.488	0.265	0.048	0.110
	2	14	0.149	0.050	0.586	0.252	0.055	0.299
Cough	0	3	0.140	0.018		0.230	0.035	
	1	27	0.162	0.048	0.155	0.256	0.058	0.327
	2	9	0.174	0.048	0.098	0.253	0.036	0.383
Pneumonitis	0	20	0.168	0.042		0.267	0.055	
	1	7	0.140	0.039	0.135	0.227	0.051	0.109
	2	12	0.168	0.056	0.998	0.247	0.041	0.265
Dermatitis	0	9	0.170	0.044		0.210	0.032	
	1	22	0.162	0.047	0.661	0.270	0.049	0.001
	2	9	0.141	0.071	0.314	0.228	0.098	0.630
Fatigue	0	4	0.171	0.046		0.224	0.057	
	1	27	0.156	0.047	0.589	0.268	0.046	0.218
	2	7	0.189	0.042	0.551	0.229	0.040	0.878
Nausea	0	30	0.157	0.048		0.249	0.053	
	1	7	0.186	0.040	0.131	0.269	0.046	0.348
	2	2	0.166	0.016	0.587	0.267	0.066	0.768
Dysphagia	0	12	0.135	0.045		0.253	0.069	
	1	13	0.168	0.037	0.061	0.244	0.044	0.690
	2	14	0.182	0.047	0.016	0.263	0.042	0.697
Cough + dysphagia	0-1	11	0.138	0.046		0.257	0.072	
	2	10	0.161	0.044	0.264	0.227	0.038	0.251
	3	15	0.170	0.042	0.083	0.239	0.098	0.583
	4	3	0.222	0.010	0.00011	0.242	0.030	0.598

^1^ CMR = complete metabolic response; ^2^ PMR = partial metabolic response; ^3^ PMD = progressive metabolic disease.

**Table 4:** Patient characteristics.

Patient ID	Age	Gender	Pathology	Stage	PTV (cm^3^)	MLD	RT/CRT	4 Weeks Beforeore or After RT	PET Response	Dyspnoea Grade	Cough Grade	Pneumonitis Grade	Dermatitis Grade	Fatigue Grade	Nausea Grade	Dysphagia Grade
GP26	59	M	SCC		N/A	11.69			N/A	1	1	0	0	0	0	0
GP27	67	M	NSCLC	T2N2M0	360	19.79	CRT: ca/pa	Before	CMR	0	1	0	2	1	1	2
GP28	69	M	AC	T2N2M0	453	15.73	CRT: ca/pa		N/A	0	1	0	0	0	0	2
GP29	49	M	AC	T4N0M0	387	8.00	CRT: cis/et	Before	N/A	1	1	0	0	2	0	1
GP30	48	F	AC	T3N3M0	493	8.75	CRT: cis/et	After	PMR	0	0	1	2	1	0	2
GP31	63	M	SCC	T3N2M1b	1081	20.45	CRT: ca/pa	After	PMD	1	2	2	1	0	0	1
GP32	64	M	SCC	T1aN1M1b	184	14.79	CRT: ca/pa	After	PMR	1	1	2	2	1	0	0
GP33	69	M	AC		251	12.29			N/A	0	1	0	0	0	0	1
GP34	60	M	AC	T2N2M1b	491	12.36	CRT: ca/pa	Before	N/A	1	1	0	2	1	1	3
GP35	70	F	SCC	T4N0M0	1074	19.86	CRT: cis/et	Before	PMD	1	1	0	1	1	0	2
GP36	75	F	AC	T2aN3M0	521	13.34	CRT: ca/pal	Before	PMR	1	2	2	1	1	0	1
GP37	78	M	AC	T2bN2M0	763	15.79	CRT: ca/pa	Before	CMR	1	1	2	2	2	0	2
GP38	68	F	AC	T3N2M1b	508	17.28	CRT: cis/et	After	N/A	1	1	0	1	1	1	2
GP39	75	M	NSCLC	T2bN3M0	913	19.81	CRT: ca/pa	Before	N/A	0	1	0	3	1	2	3
GP40	70	M	AC	T3N0M0	409	15.63	RT	Before	N/A	3	1	0	1	2	0	1
GP41	72	M	NSCLC	T1N2M0	399	16.55	CRT: ca/pa	Before	PMR	1	1	0	1	1	0	0
GP42	64	F	AC		N/A	17.39			N/A	1	1	0	0	0	0	0
GP43	67	M	SCC	T4N1M0	780	15.05			N/A	1	1	0	0	1	0	0
GP44	62	M	NSCLC	T1N2M0	415	15.36	CRT: ca/pa	Before	CMR	1	1	1	3	2	1	2
GP45	74	F	AC	T1bN3M0	718	17.10	CRT: ca/pa	Before	N/A	1	1	0	1	1	0	2
GP46	74	F	AC	T2N3M0	477	17.50	CRT: cis/et	Before	PMR	3	1	0	1	0	0	0
GP47	68	M	SCC	rT0N2M0	160	10.55	CRT: ca/pa	After	PMR	1	1	2	1	1	0	2
GP48	63	F	AC	T1N2M0	259	17.42	RT	After	CMR	2	1	2	2	1	0	0
GP49	74	M	SCC	T2aN0M0	129	8.54	RT	Before	PMD	2	1	0	1	2	0	1
GP50	78	F	NSCLC	T1N2M0	161	13.23	CRT: ca/pa	Before	CMR	1	2	2	1	1	1	1
GP51	82	M	AC	T1cM0M0	124	9.20	RT	After	PMD	2	1	0	1	1	0	0
GP52	48	M	AC	T3N2M0	338	13.86	CRT: cis/et	After	CMR	3	1	1	2	1	0	1
GP53	67	M	SCC	T1N0M0	118	15.20	CRT: cis/et	Before	CMR	2	2	2	1	2	0	2
GP54	68	F	AC	T1bN2M0	264	13.69	CRT: ca/pa	After	PMR	0	1	1	1	2	0	2
GP55	82	M	SCC	T2aN2M0	165	13.65	CRT: ca	After	CMR	0	0	0	1	1	1	0
GP56	48	M	AC	T3N2M0	502	15.04	CRT: ca/pa	After	PMR	1	1	0	1	1	0	1
GP57	68	M	AC	T3N0M0	221	12.71	CRT: ca/pa	Before	CMR	1	2	2	0	1	0	1
GP58	68	M	AC	T2aN2M0	537	16.97	CRT: ca/pa	Before	PMD	2	1	0	1	1	0	0
GP59	71	F	SCC	T4N0M0	208	11.22	CRT: ca/pa	Before	PMR	2	1	1	1	3	0	0
GP60	35	M	AC	T2bN2M1b	538	14.10	CRT: cis/et	Before	PMD	1	1	0	1	1	1	2
GP61	67	M	NSCLC	T3N2M0	804	17.45	CRT: ca/pa	After	CMR	2	1	1	0	1	0	1
GP62	80	F	AC	T1N2M0	293	18.90	CRT: ca/pa	After	CMR	2	2	2	0	2	1	2
GP63	63	F	SCC	T3N2M0	619	16.43	CRT: ca/pa		PMR	1	2	1	1	1	0	2
GP64	84	F	SCC	T4N0M0	91	5.90	RT	After	CMR	1	1	0	0	2	0	2
GP65	72	M	AC	T3N2M0	476	15.57	CRT: ca/pa	Before	PMR	0	2	1	1	1	0	0
GP66	71	M	NSCLC	T4N0M0	477	12.40	CRT: ca/pa	After	PMR	1	2	1	1	1	0	2
GP67	68	F	AC	T1aN1M0	267	14.45	RT	Before	PMD	2	1	2	1	1	0	2
GP68	80	M	SCC	T2BN2M0	281	12.59	CRT: ca/pa	After	CMR	2	2	1	1	1	0	1
GP69	71	M	SCC	T2N2M0	511	17.94	CRT: ca/pa	After	CMR	2	1	2	0	2	0	0
GP70	64	M	AC	T4N2M1b	820	15.00	CRT: ca/pa	After	N/A	1	1	0	0	1	2	1

Of the 45 patients recruited for the GalliPET VQ-RT clinical trial, 7 (data shaded grey) did not complete the full course of treatment due to disease progression (1 patient), changed to palliative treatment due to the extent of disease (1 patient), or lacked a full γ-H2AX dataset due to logistic errors, sample loss or processing failures (6 patients). The complete datasets for 38 patients were, therefore, analyzed for this translational study; M—male, F—female, SCC—squamous cell carcinoma, NSCLC—non-small cell lung cancer, AC—adenocarcinoma, ca/pa—carboplatin/paclitaxel, cis/et—cisplatin/etoposide, ca—carboplatin, N/A—not available.

## Supplementary Materials

The following are available online at https://www.mdpi.com/2072-6694/12/9/2517/s1, Figure S1: Frequency distribution histograms of *Q*- and *R* values based on the data in Table 3, Figure S2: Comparison of experimental (presented in Table S3) and calculated (as described in Text S1) foci numbers in peripheral blood mononuclear cells (PBMC) at 1 h post first RT session, Figure S3: Correlation of *Q*–values with normal tissue toxicity, Figure S4: Scatter plots of *Q*-values in subgroups of patients with different metabolic response and toxicity grades for seven toxicity criteria, Figure S5: Scatter plots of R values in subgroups of patients with different metabolic response and toxicity grades for seven toxicity criteria, Table S1: Statistics of γ-H2AX foci per cell (fpc) values in peripheral blood mononuclear cells (PBMC) collected at different time points and fixed 24 h after collection, Table S2: The values of foci kinetics parameters obtained from nonlinear regression analysis and the ratio of the mean foci number at 24 and 1 h post first radiotherapy (RT) treatment, Table S3: The results of the analysis of foci frequency distribution at 1 h post first RT, Text S1: Calculation of the number of foci induced in PBMC during local RT for NSCLC.
