# Supplementary material for: Monitoring DNA Damage and Repair in Peripheral Blood Mononuclear Cells of Lung Cancer Radiotherapy Patients
Source: Cancers (Basel). 2020 Sep 4;12(9):2517. doi: 10.3390/cancers12092517 (PMC7563254; doi:10.3390/cancers12092517)
Supplement: Supplementary file 1 [file cancers-12-02517-s001.pdf]

Supplementary Material: Monitoring DNA Damage and Repair in Peripheral Blood Mononuclear Cells of Lung Cancer Radiotherapy Patients

Pavel N. Lobachevsky, Nicholas W. Bucknell, Joel Mason, Diane Russo, Xiaoyu Yin, Lisa Selbie, David L. Ball, Tomas Kron, Michael Hofman, Shankar Siva and Olga A. Martin

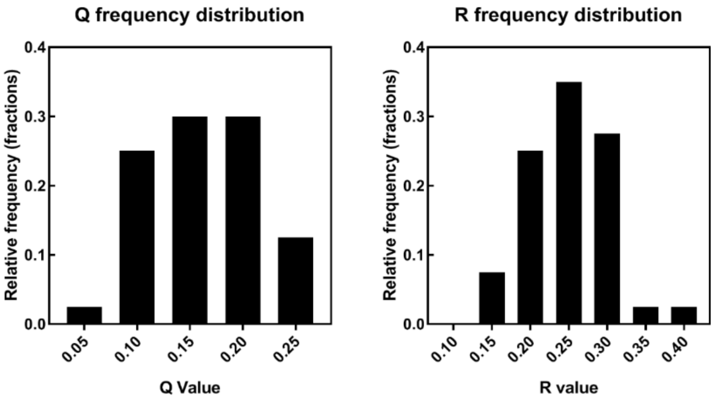

Figure S1. Frequency distribution histograms of *Q*- and *R* values based on the data in Table 3.

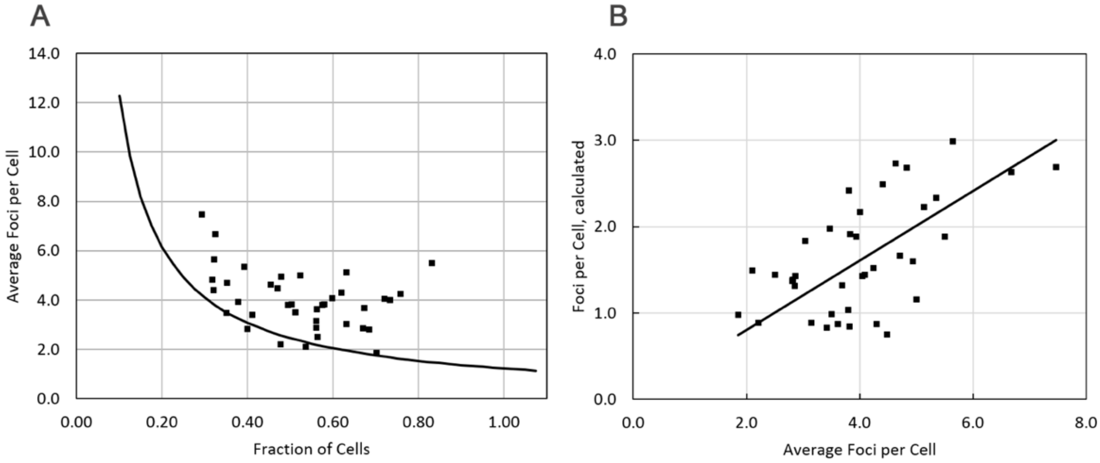

Figure S2. Comparison of experimental (presented in Table S3) and calculated (as described in Text S1) foci numbers in peripheral blood mononuclear cells (PBMC) at 1 h post first RT session. (**A**) Calculated relationship between the average foci number and the fraction of irradiated cells (solid line) and experimental data for individual patients (solid symbols). (**B**) Correlation of calculated (vertical axis) and experimental (horizontal axis) foci numbers. Symbols represent data for each patient, and solid line shows the linear regression. Pearson correlation coefficient $r = 0.604$, $p < 0.01$.

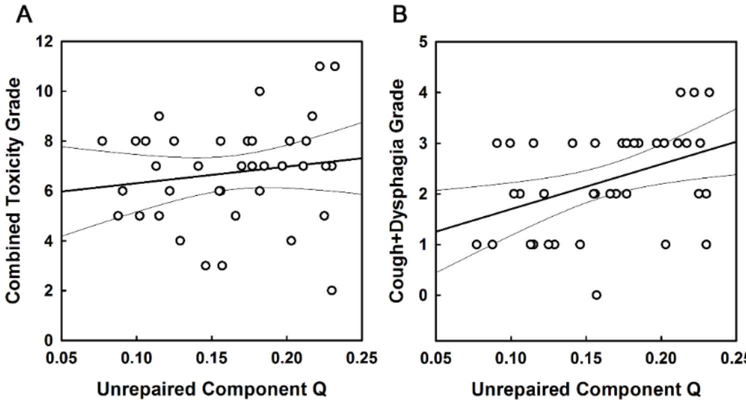

Figure S3. Correlation of *Q*–values with normal tissue toxicity. (**A**) A weak correlation of *Q*-values with a combined toxicity grade calculated as described in text ($r = 0.152$, $p = 0.36$). (**B**) Correlation of *Q*-values with a combined dysphagia/cough toxicity ($r = 0.412$, $p = 0.010$).

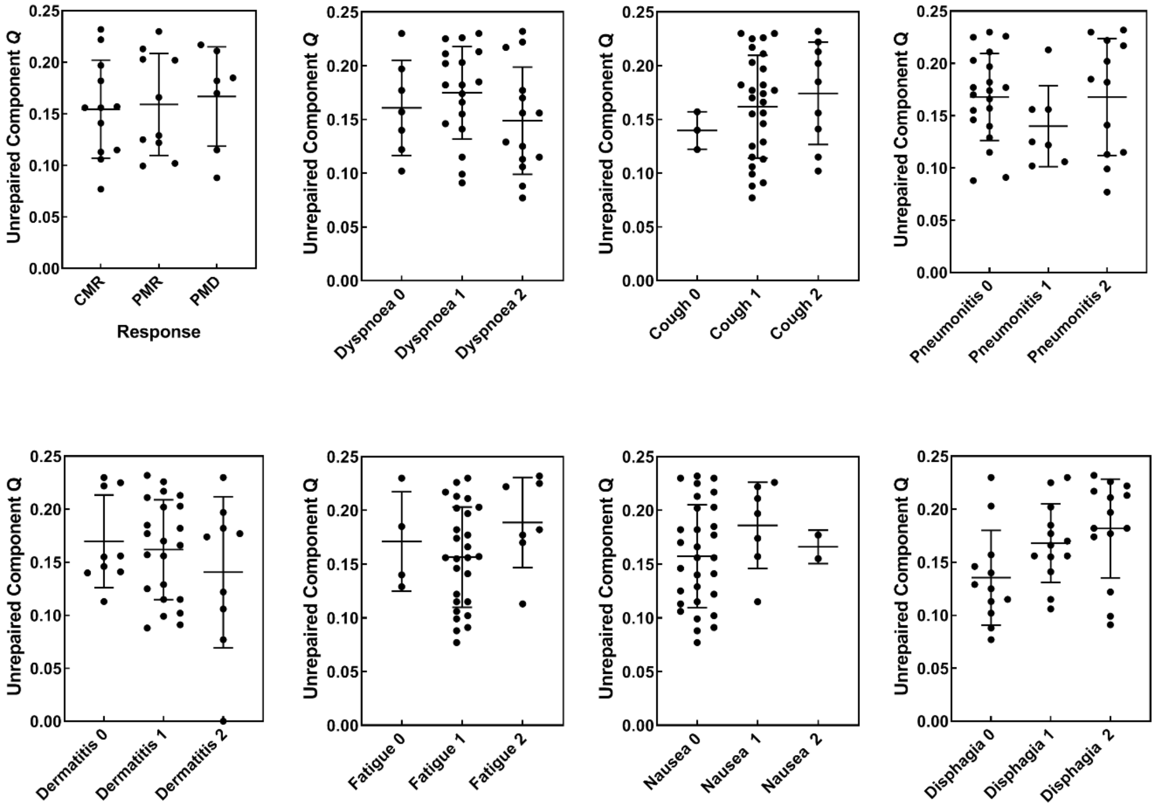

Figure S4. Scatter plots of *Q*-values in subgroups of patients with different metabolic response and toxicity grades for seven toxicity criteria.

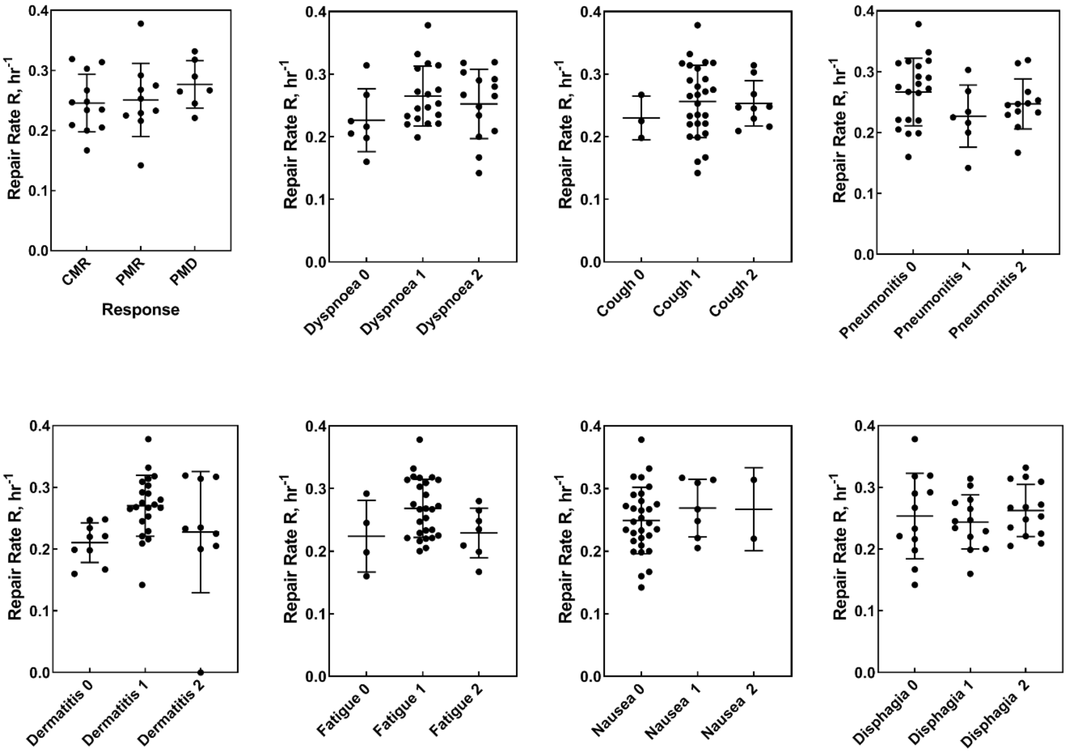

Figure S5. Scatter plots of *R* values in subgroups of patients with different metabolic response and toxicity grades for seven toxicity criteria.

Table S1. Statistics of γ-H2AX foci per cell (fpc) values in peripheral blood mononuclear cells (PBMC) collected at different time points and fixed 24 h after collection.

Time Points of Blood Collection	Baseline	1 h Post First RT	24 h Post First RT	4 Weeks Pre-RT	4 Weeks Post-RT	3 Months Post-RT
Mean Value, fpc	1.18	1.22	1.07	1.46	1.21	0.94
Standard Deviation	0.44	0.44	0.64	0.36	0.36	0.52
Number of samples	40	17	16	7	6	11
p-value [1] relative to baseline fixed immediately	0.291	0.037 *	0.759	0.207	0.091	0.624
p-value fixed early vs. fixed late	0.291	< 0.0001 *	0.948	0.017 *	0.869	0.813

[1] *p*-values are given for paired *t*-test. * Statistically significant.

Table S2. The values of foci kinetics parameters obtained from nonlinear regression analysis and the ratio of the mean foci number at 24 and 1 h post first radiotherapy (RT) treatment.

Patient ID	*Q* *	StdEr (*Q*)	*R* *	StdEr (*R*)	24 h/1 h Ratio
GP26	0.059	0.001	0.192	0.001	
GP27	0.197	0.017	0.205	0.017	0.671
GP29	0.225	0.062	0.199	0.060	0.423
GP30	0.122	0.001	0.225	0.001	0.350
GP31	0.185	0.100	0.245	0.119	0.538
GP32	0.230	0.033	0.233	0.039	0.766
GP33	0.230	0.027	0.160	0.021	
GP34	0.174	0.034	0.317	0.058	0.694
GP35	0.182	0.035	0.332	0.065	0.401
GP36	0.202	0.048	0.229	0.054	0.375
GP37	0.182	0.016	0.235	0.018	0.273
GP38	0.226	0.079	0.309	0.136	
GP39	0.177	0.040	0.314	0.068	0.251
GP40	0.177	0.025	0.280	0.035	0.567
GP41	0.203	0.059	0.378	0.138	0.323
GP42	0.140	0.113	0.198	0.099	
GP43	0.146	0.044	0.221	0.044	0.273
GP45	0.091	0.068	0.272	0.084	
GP46	0.129	0.015	0.292	0.021	0.445
GP47	0.099	0.099	0.253	0.111	0.443
GP48	0.077	0.011	0.319	0.018	0.387
GP49	0.170	0.036	0.265	0.047	0.311
GP50	0.115	0.023	0.314	0.036	0.316
GP51	0.115	0.001	0.290	0.001	0.267
GP52	0.106	0.018	0.200	0.015	0.322
GP53	0.232	0.051	0.209	0.053	0.692
GP55	0.157	0.010	0.267	0.014	0.588
GP56	0.166	0.035	0.275	0.047	0.272
GP57	0.141	0.078	0.247	0.090	0.411
GP58	0.088	0.111	0.318	0.173	0.419
GP59	0.125	0.068	0.142	0.041	0.322
GP60	0.211	0.010	0.221	0.010	0.574
GP61	0.156	0.031	0.234	0.034	0.283
GP62	0.222	0.038	0.248	0.047	0.449
GP63	0.213	0.015	0.268	0.021	0.564
GP65	0.102	0.011	0.216	0.011	0.322
GP67	0.217	0.025	0.267	0.034	
GP68	0.156	0.094	0.303	0.147	0.443
GP69	0.113	0.030	0.167	0.021	0.336
GP70	0.155	0.030	0.220	0.030	0.523

* *Q* is the fraction of unrepairable DNA damage; *R* is repair rate, the fraction of fpc per hour. Data shaded grey indicate 5 patients that lacked γ-H2AX foci counts at 1 h post first RT due to logistic errors, sample loss or processing failures. These patients were excluded from calculation of 24 h/1 h ratio and from analysis presented in Table S3.

Table S3. The results of the analysis of foci frequency distribution at 1 h post first RT.

Patient ID	Fraction of Irradiated PBMC	Standard Error of Fraction of Irradiated PBMC	Average Number of Radiation-Induced Fpc	Standard Error of Number of Radiation-Induced Fpc
GP27	0.325	0.025	6.675	0.998
GP29	0.305	0.006	3.252	0.149
GP30	0.276	0.023	4.053	0.438
GP31	0.504	0.008	3.879	0.239
GP32	0.400	0.009	2.821	0.131
GP34	0.537	0.005	2.103	0.081
GP35	0.652	0.012	4.064	0.385
GP36	0.318	0.029	4.826	0.378
GP37	0.631	0.005	3.035	0.072
GP38	0.684	0.008	2.804	0.089
GP39	0.631	0.009	5.134	0.183
GP40	0.560	0.012	2.868	0.121
GP41	0.564	0.015	2.509	0.236
GP43	0.759	0.006	4.241	0.104
GP44	0.321	0.034	4.409	0.485
GP46	0.392	0.019	5.350	0.380
GP47	0.478	0.005	2.206	0.074
GP48	0.351	0.012	3.470	0.154
GP49	0.411	0.018	3.413	0.149
GP50	0.562	0.013	3.617	0.145
GP51	0.470	0.020	4.482	0.394
GP52	0.701	0.014	1.854	0.098
GP53	0.560	0.017	3.146	0.138
GP54	0.573	0.010	5.125	0.132
GP55	0.513	0.015	3.507	0.120
GP56	0.670	0.020	2.852	0.320
GP57	0.637	0.012	3.972	0.155
GP58	0.405	0.016	4.420	0.326
GP59	0.746	0.009	3.860	0.122
GP60	0.673	0.008	3.686	0.139
GP61	0.454	0.012	4.631	0.284
GP62	0.540	0.012	4.786	0.190
GP63	0.720	0.012	4.052	0.165
GP65	0.598	0.012	4.089	0.162
GP66	0.293	0.029	7.465	1.489
GP68	0.714	0.007	4.449	0.100
GP69	0.321	0.032	5.644	0.892
GP70	0.495	0.005	3.526	0.101

Text S1. Calculation of the Number of Foci Induced in PBMC During Local RT for NSCLC

Calculation of the average fpc number induced in PBMC by irradiation of blood during local RT is a complex process that requires the knowledge of a range of static and dynamic parameters. Static parameters are the average tissue dose (tumour and/or normal tissue(s)), the irradiated volume, the volume of blood in irradiated tissue (tumour and/or normal tissue(s)) and the total blood volume. Dynamic parameters are the blood flow rate (in tumour and normal tissue) and the dose rate (or detailed timing of individual beams delivering RT fraction). Accurate consideration of all these parameters requires development of a model of blood flow in tissues of interest and superposition of the treatment plan on this model. It is a complex task

that is not subject of the present study, especially considering that accurate blood flow parameters might not be available. Therefore, here, we concentrated on calculation of a relationship of foci induction parameters that is not dependent on dynamic parameters and the value of which can also be obtained from experimental data.

First, we state that the total number of foci induced in all PBMC does not depend on dynamic parameters (blood flow and dose rate) and is proportional to the average dose and the number of PBMC in irradiated volume (which in turn is proportional to the fraction of blood, relative to the total blood volume, in irradiated volume). So, the total number of induced foci N_{tot} can be expressed as follows:

$$N_{tot} = YD\frac{Vbir}{Vb}N_{cells} \quad \text{(S1)}$$

where Y is the yield of foci per cell per Gy (approximately, 10–11 for PBMC fixed 1 h post irradiation), D is the dose of radiation, V_{bir} and V_b are the volume of blood in irradiated volume and total blood volume, respectively, and N_{cells} is the total number of PBMC in blood.

Dynamic parameters (blood flow and dose rate) will affect the fraction of irradiated PBMC (f) and the average number of foci in irradiated subpopulation (N_{av}), and although we cannot calculate these values, we can establish the relationship between them based on Equation (S1). For fraction f, the number of irradiated cells will be fN_{cells}, and we can obtain the average number of foci by dividing the total number of foci by the number of irradiated cells and substitution for N_{tot} from Equation (S1):

$$N_{av} = \frac{N_{tot}}{fNcells} = \frac{1}{f}YD\frac{V_{bir}}{V_b} \quad \text{(S2)}$$

As it follows from Equation (S2), there is an inverse proportional relationship between the average number of foci per cell and the fraction of irradiated cells.

Equation (S2) can be easy verified for extreme cases of instantaneous irradiation and indefinitely long irradiation. For instantaneous irradiation, there is no blood flow for the duration of irradiation, so all cells receive the dose D, thus, $N_{av} = YD$. The fraction of irradiated cells equals in this case to the relative irradiated blood volume $f = {V_{bir}}/{V_b}$, so Equation (S2) converts to the same:

$$N_{av} = YD \quad \text{(S3)}$$

For indefinite long irradiation (all blood/cells are mixed up and pass through irradiation volume many times), the fraction of irradiated cells f equals to 1. All cells receive the same dose D_c, and this dose is proportional to the relative time that cells are located in the irradiated volume. This relative time in turn is proportional to the relative volume of blood in the irradiation volume, so the dose is $D_c = D\,{V_{bir}}/{V_b}$ and $N_{av} = YD_c = YD\,{V_{bir}}/{V_b}$. Substitution of $f = 1$ into Equation (S2) results in the same expression:

$$N_{av} = YD\,{V_{bir}}/{V_b} \quad \text{(S4)}$$

It can be shown that in a general case of tumour/tissue(s), the term $D\frac{V_{bir}}{V_b}$ is expressed as a sum of similar terms for each tissue that is subjected to irradiation.

The following parameters can be used to calculate the relationship between the average foci number and the fraction of irradiated cells:

(1) Blood volume—5000 mL
(2) Pulmonary blood volume—500 mL
(3) Average tumour volume—500 mL
(4) Lung volume—3000 mL
(5) Tumour dose—2 Gy
(6) Lung dose—0.49 Gy (one fraction)

Foci yield-15 foci/Gy/cell (although the reported yield of foci in PBMC varies in the range 9–12 foci/Gy/cell, for low doses up to 100 mGy, higher yield is reported).

We also assumed that the ratio of blood volume/tissue volume is similar for tumour and lung.

Based on these parameters, the dose contributions $D\frac{V_{bir}}{V_b}$ are 49 and 33 mGy for lung and tumour, respectively. The calculated relationship between the average foci number and the fraction of irradiated cells is demonstrated in Figure S2A as a solid line. Data points show the results for individual patients presented in Table S3. Figure S2B demonstrates correlation between the average for each patient foci number (data from Table S3) and foci number calculated using Equation (S2) based on individual parameters (tumour volume and MLD) for each patient.
